# Identifying common patterns of health services use: a longitudinal study of older Swiss adults' care trajectories

**DOI:** 10.1186/s12913-022-08987-z

**Published:** 2022-12-26

**Authors:** Leonard Roth, Laurence Seematter-Bagnoud, Marie-Annick Le Pogam, Julien Dupraz, Juan-Manuel Blanco, Yves Henchoz, Isabelle Peytremann-Bridevaux

**Affiliations:** Department of Epidemiology and Health Systems, Centre for Primary Care and Public Health (Unisanté), University of Lausanne, 10 Route de La Corniche, 1010 Lausanne, Switzerland

**Keywords:** Healthcare utilization, Trajectories, Sequence analysis, Cohort study, Community-dwelling older adults

## Abstract

**Background:**

Population ageing puts pressure on health systems initially designed to handle acute and episodic illnesses. Segmenting an ageing population based on its healthcare utilization may enable policymakers to undertake evidence-based resource planning. We aimed to derive a typology of healthcare utilization trajectories in Swiss older adults.

**Methods:**

Our work used data from the Lc65 + study, a population-based cohort of individuals aged 65 to 70 years at enrolment. The dimensions of healthcare utilization considered were ambulatory care, emergency care, hospitalizations, professional home care and nursing home stay. We applied the Sequence Analysis framework, within which we quantified the variation between each multidimensional pair of sequences, implemented a clustering procedure that grouped together older persons with similar profiles of health services use, and characterized clusters of individuals using selected baseline covariates.

**Results:**

Healthcare utilization trajectories were analysed for 2271 community-dwelling older adults over a period of 11 years. Six homogeneous subgroups were identified: constant low utilization (83.3% of participants), increased utilization (4.9%), late health deterioration (4.4%), ambulatory care to nursing home (1.5%), early fatal event (3.8%) and high ambulatory care (2.1%). Associations were found between cluster membership and age, sex, household composition, self-perceived health, grip strength measurement, comorbidities, and functional dependency.

**Conclusions:**

The heterogeneous healthcare utilization profiles can be clustered into six common patterns. Different manifestations of functional decline were apparent in two distinct trajectory groups featuring regular home care use. Furthermore, a small proportion of individuals with a unique set of characteristics was related to the highest levels of ambulatory and emergency care use. New research avenues are outlined to investigate time-varying effects of health factors inside the clusters containing most unfavourable outcomes.

**Supplementary Information:**

The online version contains supplementary material available at 10.1186/s12913-022-08987-z.

## Key points

- Long-term healthcare utilization trajectories in older populations have received little attention so far.
- Six distinct patterns of health services use were identified in a broad population of older adults; each trajectory group was associated with a core set of socio-demographic and health factors.
- While most study participants had comparatively few interactions with the health system, specific subpopulations experiencing functional dependency, intensive utilization and early deaths were highlighted.

## Why does this paper matter?

A thorough analysis of older persons’ healthcare utilization trajectories between age 65 and 80 is essential to understand their healthcare needs and adapt health services accordingly.

## Introduction

The population is ageing at an unprecedented pace around the world [1]. In high-income countries such as Switzerland, one in five people is currently aged over 65 years, and it is predicted that this proportion will exceed one in four people by 2040 [2]. While older Swiss can benefit from one of the highest life expectancies in the world [2], between half and three-quarters of people over age 65 living at home report multiple chronic illnesses [3, 4]. There is thus a large and increasing number of individuals with evolving care needs, which causes substantial organisational and financial pressure on healthcare systems that were initially designed to handle acute and episodic illnesses [5]. This challenge is complex and multi-faceted, but research can help by studying holistically how older adults interact with the health system [5, 6]. In particular, segmenting an ageing population based on its healthcare utilization may enable policymakers to undertake evidence-based healthcare resource planning [7, 8].

Studies based on this approach have often been limited to a transversal segmentation of health services use, which does not allow for the observation of transitions between care providers that emerge when considering the evolution in time of healthcare utilization [8, 9]. This longitudinal aspect has been modelled in a study investigating whether frailty was associated with higher use of health services among the older population, where the statistical associations were estimated as average effects over the entire sample [10]. When exploring a heterogeneous population like community-dwelling older adults, it has been argued that public health research would benefit from a more refined modelling of the variation present in the longitudinal data by identifying not one but multiple typical trajectories [11, 12]. These homogeneous subgroups with distinct healthcare needs can then constitute the basis for targeted care delivery strategies [13]. Several studies have brought valuable insights on typical trajectories of health services use but were often restricted to a specific condition (heart failure [14]; epilepsy [15]; hypertension [16]; multiple sclerosis [17]) and/or to a single category of care events (e.g., hospitalizations as in [14, 15, 18]). There is thus limited literature on the multidimensional patterns of care in an ageing population, which is a vital element of the integrated care concept promoting a coordinated approach to healthcare delivery versus a fragmented one [19, 20].

Our objectives are (1) to derive a typology of healthcare utilization trajectories in a cohort of older adults, and (2) to outline socio-demographic and health characteristics associated with each homogeneous subgroup.

## Methods

### Study design and population

The Lc65 + study is an ongoing population-based cohort of older adults living in the city of Lausanne, Switzerland, primarily investigating the manifestations, determinants, and outcomes of frailty [21]. A randomly selected sample of community-dwelling older adults aged 65 to 70 years was contacted in three distinct recruitment waves (2004, 2009, and 2014 cohorts, respectively) [22]. Approximately half the eligible subjects accepted to be enrolled and it has been shown that all three samples were representative of the target population for socio-demographic characteristics [23]. Lc65 + participants are asked to fill out a yearly postal questionnaire and undergo an interview with a medical examination every three years, allowing to collect information in a wide range of domains such as physical and mental health, economic status, social network, healthcare utilization [22].

Our work used data from the first and second cohorts, which included 1564 and 1489 participants, respectively, at baseline (Supplementary Figure S1). Data from the third cohort were excluded as the follow-up duration was deemed too short. The last five years of follow-up for the first cohort were not included in order to group individuals from this cohort together with the ones from the second cohort, and hence derive healthcare utilization trajectories on 11 years of follow-up. Thus, the information was collected prospectively from 2005 to 2015 (cohort 1) or from 2010 to 2020 (cohort 2) on individuals aged up to 81 years in 2015 and 2020, respectively. We required at least six observations over the 11 years of follow-up for an individual to be considered in the analysis. This criterion was set up against other options tested in a sensitivity analysis to optimise the length and completeness of healthcare utilization trajectories while minimising the number of individuals lost to attrition. The final study sample comprised 2271 older adults out of the 3053 recruited in the first and second waves.

### Variables

#### Outcome measures

The different dimensions of healthcare utilization considered in the Lc65 + cohort are ambulatory care (number of physician's appointments), emergency care (number of emergency consultations), hospitalizations (overnight stays), professional home care and nursing home stay (at least one night). This information is self-reported every year and the five dimensions of healthcare utilization were categorized according to the frequency of use.

#### Independent factors

A range of a priori relevant covariates were selected to characterize the healthcare utilization profiles, based on existing literature and the investigators' domain-specific knowledge. These potential determinants of health services use were collected at baseline through the postal questionnaire or the first interview; they are thematically listed below.

Recruitment wave (first or second); demographic factors: age (65 to 70 years), sex (female/male), children (yes/no), marital status (single/married/separated/widowed), living alone (yes/no), born in Switzerland (yes/no); socio-economic factors: educational level (basic/technical/secondary/higher), financial hardship (yes/no), health insurance subsidy (yes/no), supplementary private hospital insurance (yes/no); behavioural factors: rarely leaving the house (yes/no), physical activity (normal/low), alcohol consumption (never/occasional/frequent), smoking status (current/former/never); health factors: BMI (normal/underweight/overweight/obese), measured grip strength (normal/low from Fried’s frailty criteria), measured cognitive function (normal/low based on MMSE < 24), number of diagnosed chronic illnesses (0/1/2 + among hypertension, hypercholesterolaemia, cardio − /cerebrovascular disease, diabetes, pulmonary disease, osteoporosis, osteoarthritis or other arthritis, malignancy, Parkinson’s disease, Alzheimer’s disease), types of medications used (0/1–4/ 5 +), self-perceived health (good/average/bad), dependency in Basic Activities of Daily Living (none/difficulty but no help/difficulty with help), dependency in Instrumental Activities of Daily Living (none/difficulty but no help/difficulty with help).

### Statistical analysis

#### Dissimilarity measure

We followed the Sequence Analysis (SA) framework to derive a typology of healthcare utilization trajectories [24]. SA is a statistical method that models the longitudinal data as individual sequences of categorical states separately for all care settings and has been demonstrated to be particularly suitable for exploratory analyses of care trajectories [25, 26]. To quantify the variation between each multidimensional pair of sequences, we applied a dissimilarity measure that summarizes the discrepancy across trajectories into a numerical matrix [27]. As both the timing and sequencing of the healthcare utilization states were important in our study, we picked Optimal Matching (OM) for the dissimilarity measure [28]. OM computes the distance between any two sequences as the minimum total cost of transforming one sequence into the other by means of substitutions or insertion-deletions (indel) of single states [29]. In our work, substitution costs were user-defined for each dimension of healthcare utilization, with levels of utilization that were further apart being assigned a higher substitution cost. We set indel costs to half the maximum substitution costs in order to give equal importance to timing and sequencing. We applied the multichannel version of OM to our multidimensional trajectories, where sequences are compared and costs additively combined at each time point when measuring dissimilarities (local interdependence) [30]. Each channel was given a weight proportional to the size of its specific indel cost in the final measure to avoid having one dimension of health services use dominating the others in the distance matrix. Different options for the dissimilarity measures were tried in a sensitivity analysis. An example of the application of our favoured dissimilarity measure is demonstrated in the supporting information.

#### Clustering

A clustering procedure was implemented based on the distance matrix; this allowed grouping of older people with similar profiles of health services use. Different methods are available for this step of a SA, including partitioning around medoids, agglomerative clustering with complete linkage, and agglomerative clustering with Ward linkage [31]. To discriminate between clustering procedures, we used the average silhouette value, which measures for each cluster the cohesion inside of it and the separation from all others [32]. The optimal method in our case was hierarchical clustering with complete linkage, where the distance between any two clusters evaluated during the agglomerative step is the maximum distance between any two multidimensional sequences that are in these clusters. Hierarchical clustering makes it straightforward to compare solutions with different numbers of clusters. Increasing the number did not distinctly decrease the silhouette value. Therefore, we set the criterion that all clusters contained at least ~ 1% of the participants, i.e., at least 25 of them. This ensured a more meaningful cluster characterization.

#### Cluster characterization

Clusters of individuals were first characterized in a descriptive way using the selected baseline covariates. Bivariate associations with cluster membership were evaluated with chi-squared tests for categorical covariates and ANOVA for the age. Then, we used a multivariable multinomial logistic regression model to adjust for potential confounders. All associations that were found to be significant at a 95% confidence level in the bivariate setting were initially included in the multivariable setting. However, there were concerns of collinearity across effects because of the numerous covariates featured in the multinomial regression. To further improve the model's goodness of fit and parsimony, we operated a backwards stepwise variable selection, where covariates were discarded one by one as indicated by the Akaike Information Criterion. The selection procedure was stopped once removing variables from the regression did not improve the information criterion anymore. Thus, the final model allowed estimating independent associations between a set of optimal covariates and cluster membership. The estimated effects corresponded to odds ratios of a participant being assigned to a cluster relative to the reference cluster, which in our case was the trajectory group containing the most individuals. A polynomial function of the age—as well as interactions between a priori relevant covariates—were considered but did not pass the variable selection test.

#### Missing data

There were several missing observations in the outcome measures. Some were missing at random while others were missing not at random (i.e. nonignorable nonresponses) due to individuals either being too unwell to participate in the Lc65 + study (incapacitated) or deceased. These two states were assigned specific categories in the outcome and all missing observations were accounted for in the dissimilarity measure through explicit modelling. When setting substitution costs, deaths were the furthest apart from no utilization, too unwell to participate was equivalent to the highest utilization level, and random nonresponses were equidistant to all other states. Furthermore, missing values in the independent factors were grouped in a corresponding added category when they represented more than 0.5% of the observations and not included in the analysis otherwise.

All analyses were performed using the statistical software R v4.0.3, with the help of packages *TraMineR *[27], *WeightedCluster *[31] and *nnet* among others*.*

## Results

**Fig. 1:**
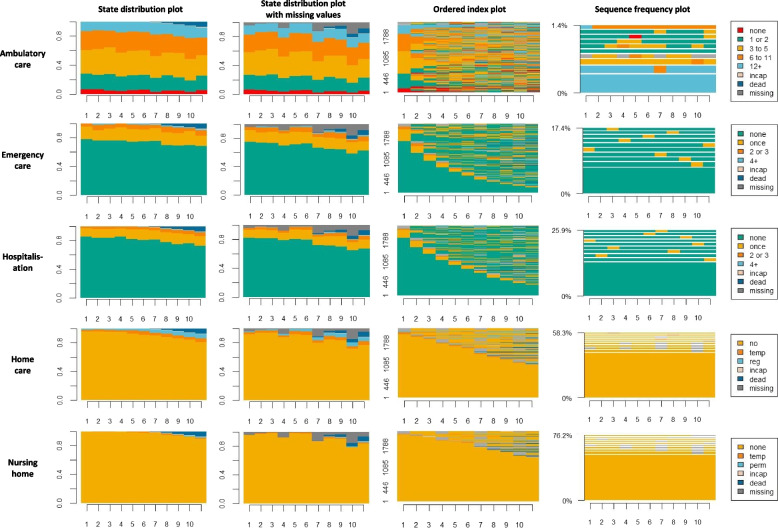
Exploratory sequence analysis for the 2271 selected older adults. X-axis is years since entry into the study. State distribution plots represent the cumulative frequency of participants in each state at a given time. Index plots represent the 2271 individual trajectories. Sequence frequency plots represent the ten most frequent trajectories and how often they occur. ‘Incap’ stands for incapacitated, ‘temp’ for temporary, ‘reg ‘ for regularly and ‘perm’ for permanent

Our sample of 2271 older adults was in average 67.9 years old at enrolment (median: 68 years old), with 60.7% of women. About one-third (36.9%) lived alone, about one in ten (12.6%) persons faced financial hardship, 62.4% had two or more chronic conditions but 70.5% reported that their health was good (Tables 1 and 2). Figure 1 depicts the healthcare utilization trajectories as categorical sequences over an 11-year timeframe. All five dimensions of health services use considered in the study are explored in parallel. In the state distribution plots, the cumulative frequency of self-reported healthcare utilization states is displayed for each study year. The distribution stays relatively stable, with few deaths appearing toward the end of follow-up. Random nonresponses are included in the figure's second column. They correspond to 5.9% of all observations. The index plots show all individual sequences, featuring a large variety of trajectories. This result is confirmed in the sequence frequency plots, at least for ambulatory care where less than 0.5% of the sequences are identical. This proportion increases markedly in the other care settings as utilization levels decrease, with, for instance, more than 60% of the participants reporting no use of nursing home facilities throughout the follow-up.

**Table 1:** General information, demographic and socioeconomic characteristics of study participants, by cluster

		**Whole sample**	**LHU**	**IHU**	**LHD**	**AC2NH**	**EFE**	**HAC**	***p*-value**
**Total**	N	2271	1893	111	101	33	86	47	
	%	100	83.4	4.9	4.4	1.5	3.8	2.1	
**Recruitment wave**	1	50.9%	50.3%	55.9%	52.5%	57.6%	47.7%	57.4%	0.673
	2	49.1%	49.7%	44.1%	47.5%	42.4%	52.3%	42.6%	
**Age (years)**	Mean	67.9	67.8	68.2	68	68.8	67.9	68.1	0.001
	SD	1.4	1.4	1.4	1.5	1.2	1.4	1.5	
**Sex**	Female	60.7%	60.9%	64.9%	50.5%	60.6%	50.0%	83.0%	0.002
	Male	39.3%	39.1%	35.1%	49.5%	39.4%	50.0%	18.0%	
**Children**	No	21.1%	20.9%	18.0%	20.8%	30.3%	27.9%	19.1%	0.374
	Yes	78.3%	78.6%	82.0%	77.2%	69.7%	70.9%	80.9%	
	missing	0.5%	0.5%	0.0%	2.0%	0.0%	1.2%	0.0%	
**Marital status**	Single	12.2%	11.8%	13.5%	11.9%	24.2%	16.3%	6.4%	< 0.001
	Married	56.1%	57.7%	49.5%	54.5%	18.2%	50.0%	46.8%	
	Separated	18.8%	17.9%	22.5%	22.8%	30.3%	19.8%	29.8%	
	Widowed	12.5%	12.3%	11.7%	10.9%	24.2%	14.0%	17.0%	
	missing	0.4%	0.3%	2.7%	0.0%	3.0%	0.0%	0.0%	
**Living alone**	No	62.8%	64.6%	55.0%	61.4%	27.3%	58.1%	48.9%	0.001
	Yes	36.9%	35.1%	45.0%	38.6%	72.7%	41.9%	51.1%	
	missing	0.3%	0.3%	0.0%	0.0%	0.0%	0.0%	0.0%	
**Born in** **Switzerland**	No	26.4%	25.7%	33.3%	35.6%	15.2%	26.7%	25.5%	0.006
	Yes	73.5%	74.2%	66.7%	64.4%	84.8%	73.3%	72.3%	
	missing	0.1%	0.1%	0.0%	0.0%	0.0%	0.0%	2.1%	
**Educational level**	Basic	20.1%	19.1%	30.6%	25.7%	18.2%	19.8%	27.7%	0.040
	Technical	39.5%	39.7%	41.4%	36.6%	42.4%	40.7%	29.8%	
	Secondary	24.7%	25.5%	13.5%	19.8%	27.3%	25.6%	25.5%	
	Higher	15.0%	15.2%	11.7%	16.8%	9.1%	14.0%	17.0%	
	missing	0.7%	0.5%	2.7%	1.0%	3.0%	0.0%	0.0%	
**Financial hardship**	No	83.0%	84.0%	76.6%	82.2%	63.6%	82.6%	72.3%	< 0.001
	Yes	12.6%	11.3%	21.6%	11.9%	30.3%	17.4%	27.7%	
	missing	4.4%	4.8%	1.8%	5.9%	6.1%	0.0%	0.0%	
**Insurance** **subsidy**	No	82.7%	84.3%	68.5%	77.2%	66.7%	77.9%	80.9%	< 0.001
	Yes	16.1%	14.4%	31.5%	21.8%	33.3%	19.8%	17.0%	
	missing	1.3%	1.3%	0.0%	1.0%	0.0%	2.3%	2.1%	
**Supplementary insurance**	No	52.2%	51.5%	58.6%	57.4%	75.8%	44.2%	53.2%	0.006
	Yes	46.4%	47.3%	36.9%	40.6%	21.2%	53.5%	46.8%	
	missing	1.5%	1.2%	4.5%	2.0%	3.0%	2.3%	0.0%	

*Legend:* Bivariate relationships are evaluated with chi-squared tests (ANOVA for the age). *LHU* cluster is low healthcare utilization, *IHU* is increased healthcare utilization, *LHD* is late health deterioration, *AC2NH* is ambulatory care to nursing home, *EFE* is early fatal event and *HAC* is high ambulatory care

**Table 2:** Behavioural and health characteristics of study participants, by cluster

		**Whole sample**	**LHU**	**IHU**	**LHD**	**AC2NH**	**EFE**	**HAC**	***p*-value**
**Total**	**N**	**2271**	**1893**	**111**	**101**	**33**	**86**	**47**	
**Rarely** **leaving the house**	No	96.8%	97.4%	98.2%	97.0%	72.7%	90.7%	100.0%	< 0.001
	Yes	2.7%	2.2%	1.8%	3.0%	21.2%	9.3%	0.0%	
	missing	0.5%	0.5%	0.0%	0.0%	6.1%	0.0%	0.0%	
**Physical** **activity**	Normal	92.0%	93.0%	94.6%	89.1%	66.7%	82.6%	87.2%	< 0.001
	Low	6.5%	5.7%	3.6%	7.9%	24.2%	17.4%	10.6%	
	missing	1.5%	1.3%	1.8%	3.0%	9.1%	0.0%	2.1%	
**Alcohol** **consumption**	Never	10.9%	10.3%	14.4%	8.9%	27.3%	11.6%	17.0%	0.009
	Occasional	58.4%	58.6%	55.9%	60.4%	45.5%	54.7%	66.0%	
	Frequent	29.1%	29.5%	27.0%	28.7%	18.2%	32.6%	17.0%	
	missing	1.7%	1.5%	2.7%	2.0%	9.1%	1.2%	0.0%	
**Smoking status**	Current	16.5%	14.9%	24.3%	24.8%	21.2%	29.1%	14.9%	0.010
	Former	39.3%	39.4%	38.7%	40.6%	39.4%	39.5%	36.2%	
	Never	43.7%	45.2%	36.9%	33.7%	39.4%	30.2%	48.9%	
	missing	0.5%	0.5%	0.0%	1.0%	0.0%	1.2%	0.0%	
**BMI**	Normal	33.0%	34.1%	27.9%	21.8%	21.2%	38.4%	25.5%	< 0.001
	Underweight	1.3%	1.3%	0.9%	2.0%	3.0%	1.2%	0.0%	
	Overweight	39.0%	39.7%	38.7%	37.6%	18.2%	33.7%	38.3%	
	Obese	22.2%	20.2%	30.6%	33.7%	45.5%	26.7%	34.0%	
	missing	4.5%	4.8%	1.8%	5.0%	12.1%	0.0%	2.1%	
**Grip strength**	Normal	83.8%	85.2%	77.5%	81.2%	54.5%	77.9%	76.6%	< 0.001
	Low	11.3%	9.6%	18.9%	13.9%	33.3%	20.9%	23.4%	
	missing	4.9%	5.2%	3.6%	5.0%	12.1%	1.2%	0.0%	
**Cognitive function**	Normal	91.6%	91.9%	90.1%	84.2%	90.9%	95.3%	95.7%	0.021
	Low	3.7%	3.3%	7.2%	8.9%	0.0%	3.5%	2.1%	
	missing	4.7%	4.9%	2.7%	6.9%	9.1%	1.2%	2.1%	
**Number of diagnosed chronic** **illnesses**	0	13.7%	14.5%	9.0%	10.9%	0.0%	16.3%	0.0%	< 0.001
	1	23.8%	24.8%	18.9%	25.7%	12.1%	19.8%	4.3%	
	2 +	62.4%	60.4%	72.1%	63.4%	84.8%	64.0%	95.7%	
	missing	0.2%	0.2%	0.0%	0.0%	3.0%	0.0%	0.0%	
**Types of medications used**	0	19.3%	20.5%	13.5%	17.8%	9.1%	16.3%	0.0%	< 0.001
	1–4	74.4%	74.4%	73.0%	72.3%	66.7%	77.9%	83.0%	
	5 +	4.9%	3.7%	11.7%	8.9%	21.2%	5.8%	17.0%	
	missing	1.4%	1.4%	1.8%	1.0%	3.0%	0.0%	0.0%	
**Self-** **perceived health**	Good	70.5%	74.6%	51.4%	64.4%	24.2%	53.5%	25.5%	< 0.001
	Av	25.8%	22.9%	42.3%	30.7%	48.5%	38.4%	55.3%	
	Bad	3.5%	2.3%	6.3%	5.0%	24.2%	8.1%	19.1%	
	missing	0.2%	0.2%	0.0%	0.0%	3.0%	0.0%	0.0%	
**Difficulties BADLs**	None	93.6%	95.1%	87.4%	91.1%	54.5%	90.7%	85.1%	< 0.001
	Yes without help	4.8%	3.9%	8.1%	5.0%	30.3%	7.0%	12.8%	
	Yes with help	0.7%	0.3%	0.9%	4.0%	12.1%	2.3%	2.1%	
	missing	0.9%	0.8%	3.6%	0.0%	3.0%	0.0%	0.0%	
**Difficulties IADLs**	None	90.6%	93.2%	80.2%	83.2%	36.4%	86.0%	72.3%	< 0.001
	Yes without help	5.3%	4.2%	11.7%	10.9%	18.2%	4.7%	17.0%	
	Yes with help	3.3%	1.8%	7.2%	5.9%	39.4%	9.3%	10.6%	
	missing	0.8%	0.8%	0.9%	0.0%	6.1%	0.0%	0.0%	

*Legend:* Bivariate relationships are evaluated with chi-squared tests. LHU cluster is low healthcare utilization, IHU is increased healthcare utilization, LHD is late health deterioration, AC2NH is ambulatory care to nursing home, EFE is early fatal event and HAC is high ambulatory care. B/IADLs = Basic/Instrumental Activities of Daily Living

**Fig. 2:**
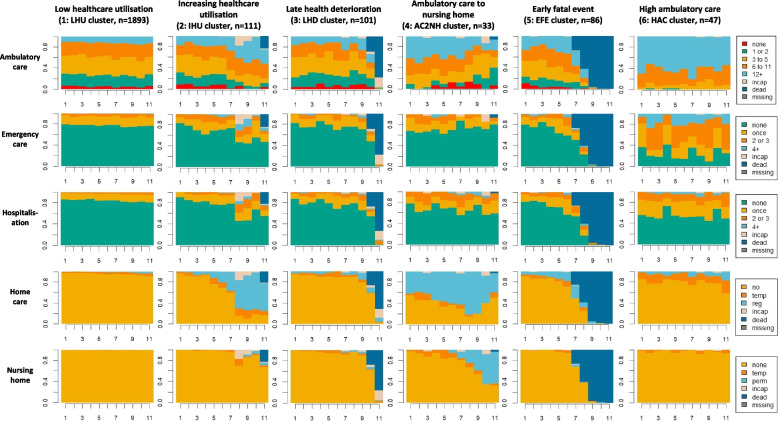
State distribution plots for the six clusters of healthcare utilization trajectories in the sample of older adults. X-axis represents years since entry into the study and y-axis is the cumulative proportion of participants in each state over time. ‘Incap’ stands for incapacitated, ‘temp’ for temporary, ‘reg’ for regularly and ‘perm’ for permanent

Figure 2 describes the six trajectory groups obtained from a hierarchical clustering procedure based on a multichannel OM dissimilarity measure. The first cluster (83.3% of participants) is the largest and contains individuals with constant low utilization throughout the study duration. Specifically, participants in this cluster almost never report staying at a nursing home or receiving formal home care, and report only sporadic emergency care use or hospitalisations. Ambulatory care use is more diverse but corresponds to moderate utilization compared to the general population. Individuals in the second cluster (4.9%) start with low healthcare utilization as well, but experience increased utilization of health services during follow-up, especially in terms of formal home care. Individuals in the third cluster (4.4%) start with a low healthcare utilization again and maintain it until late in the study, where a large proportion of them die or become too unwell to participate. The fourth cluster (1.5%) consists of participants with relatively high ambulatory care use at the start, before shifting to nursing home stay toward the end of follow-up. Compared to the other trajectory groups, this one exhibits particularly high levels of hospitalizations and home care. Individuals in the fifth cluster (3.8%) all experience an early death during the study, with relatively low healthcare utilization beforehand. Finally, participants in the sixth cluster (2.1%) report by far the highest numbers of ambulatory care visits and emergencies, but low home care use. While fluctuations remain inside these clusters, they each represent specific utilization patterns and reveal thus homogeneous subgroups inside a largely heterogeneous population.

**Fig. 3:**
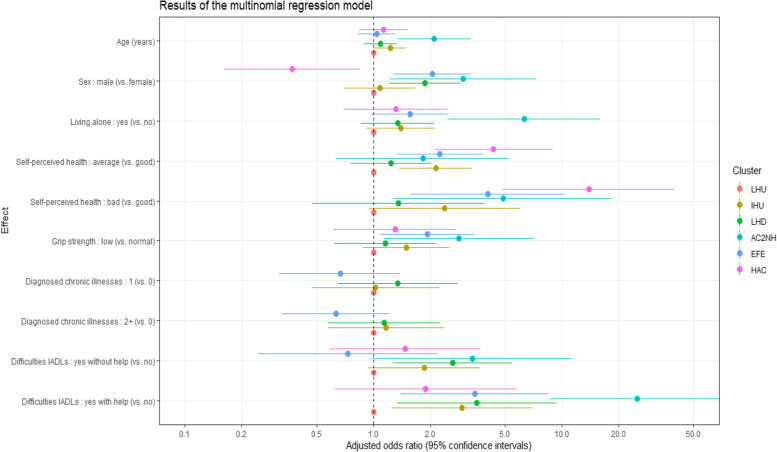
Selected results (*n* = 2256) from the multinomial regression model with the low healthcare utilization cluster as reference. IHU stands for increased healthcare utilization, LHD for late health deterioration, AC2NH for ambulatory care to nursing home, EFE for the early fatal event and HAC for high ambulatory care

Tables 1 and 2 characterize the whole sample as well as the identified clusters through a wide range of information collected at baseline. Interestingly, the recruitment wave (2004 or 2009) is not associated with cluster membership. Other associations between the healthcare utilization typology and demographic, socio-economic, behavioural, as well as health factors are apparent in this bivariate setting. Figure 3 characterizes the typology through a parsimonious set of determinants that are independently associated with cluster membership. In this multivariable setting, all effects are estimated after adjustment for the other variables in the model. As indicated in Fig. 3, being older was associated with higher odds of membership to the two clusters with strong home care use. Being male was associated with the clusters featuring a high rate of deaths during follow-up and being female was associated with membership to the high ambulatory care cluster. Living alone was associated with membership to the cluster with increasing nursing home use. Poorer self-perceived health was associated with higher odds of experiencing intensive healthcare utilization trajectories. Low grip strength measurement was associated with the “ambulatory care to nursing home” and “early fatal event” clusters. No individuals in the highest utilization clusters (fourth and sixth) had zero diagnosed chronic conditions at baseline, so the corresponding odds ratios could not be plotted. However, Table 2 clearly shows that living with more comorbidities characterizes the older adults in these two clusters. Finally, functional dependency was associated with trajectories involving high formal home care use. Estimated model coefficient values are reported in Supplementary Table S2.

### Sensitivity analysis

Participants included in the analysis are compared to those excluded in Supplementary Table S1. Exclusions were due to incomplete trajectories, which is linked to distinct characteristics for the two sets of participants. Consequently, our main typology underestimated to some extent the proportion of participants in clusters with unfavourable outcomes. However, the typical trajectories were robust to fluctuations in the selection criterion. Furthermore, the clustering procedure led to similar results if the dissimilarity measure's components were modified incrementally.

## Discussion

This work explored common patterns of health services use in a population of community-dwelling older persons. Six clear and distinct trajectory groups were revealed as a result, consistent with previous research highlighting the wide variation in health services use among older adults [9]. Most participants had comparably few interactions with the healthcare system. Thus, part of our analysis' appeal is to address the heterogeneity present in the longitudinal data and to identify as well as characterize the divergent trajectories that entailed higher utilization levels.

The largest subpopulation (LHU cluster in Fig. 2) comprised individuals with constant low healthcare utilization and no deaths during follow-up, which are favourable outcomes both from the patients' and the health systems' perspective. This finding is original per se as there is limited literature on long-term utilization trajectories followed by a general or not acutely ill population of older adults. The pattern (IHU cluster) most similar to the largest and reference cluster was notably associated with functional dependency, which is in line with the marked increase in home care use observed toward the end of follow-up. Another subpopulation (AC2NH cluster) featured trajectories with intensive home care use from the start. It had a distinctive characterization, including a higher prevalence of functional dependency at baseline. Thus, functional decline varies in both its manifestation and its association with healthcare utilization between these two clusters, which is consistent with previous research emphasizing the heterogeneity in functional ageing [33].

Two patterns featured a high proportion of deaths during follow-up, either in the last two years (LHD cluster) or earlier on (EFE cluster). Both were associated with being male, which can be related to the well-documented gender gap in life expectancy [34]. In addition, the trajectory group with early deaths was also associated with low baseline grip strength measurements and worse self-perceived health – both found to be significant predictors of all-cause mortality in previous studies [35, 36] – but, interestingly, not with a higher prevalence of diagnosed chronic diseases. In fact, both these clusters featured trajectories with average to low healthcare utilization at the start. A possible explanation is that some deaths are related to accidents and not illnesses, or sudden events such as acute pneumonia. Alternatively, it could be a sign of under-utilization of effective medical services. In this scenario, patients may receive suboptimal care due to several factors such as non-adherence, which ultimately leads to worse health outcomes [37].

Finally, two patterns of intensive health services use were identified – one with constant high ambulatory care (HAC cluster) and one, previously discussed, with a transition to nursing home during follow-up (AC2NH cluster). Both corresponded to individuals with comorbidities and a poorer self-perceived health, which is not surprising as it reflects the impact of chronic conditions on healthcare utilization [38]. Notable differences were that the constant pattern was associated with being female while the transition one was associated with low grip strength. Previous work had investigated gender disparities in healthcare utilization, finding that the higher number of physician visits observed for women was solely due to their greater health needs [39]. On the other hand, grip strength measurement is a proxy for frailty, a status that has been shown to increase the use of healthcare before the onset of disability [10]. These last two subpopulations contained relatively few participants, which is consistent with previous research on 'high need high cost' individuals indicating that a high proportion of healthcare costs are related to a small proportion of patients [7].

Interestingly, while other studies point out the impact of socio-economic factors on health services use, be it in the context of healthcare needs [40] or for diabetes patients [41], we did not find such associations in our work after adjustment for all measured confounders. This may be a specificity of the Swiss setting, where coverage is ensured through mandatory health insurance, with subsidies for people on low income [42].

We performed for this project a full Sequence Analysis, a methodology that has risen in popularity among health services researchers in recent years [17, 25, 26, 41], but that had previously seen decades of development in social sciences, where many phenomena of interest can be described as sequences of categorical states [44]. The recent adoption in the public health literature has been driven by the exponential increase in longitudinal data available to researchers [12], and by the necessity to identify homogeneous subgroups in heterogeneous populations for real-life studies [11]. This last aspect is apparent in our analysis, where small but distinct clusters mirroring the healthcare needs of specific subpopulations were highlighted.

Nevertheless, our study results need to be interpreted considering the following limitations. First, the outcome measures were self-reported, which has been linked to underreporting of healthcare utilization—particularly for physician visits and high users [43]. However, our main findings are robust to moderate biases in utilization rates, and analysing survey data enable us to characterize the clusters in a more comprehensive manner than what would be possible with most administrative databases. Second, early loss to follow-up or repeated temporary nonresponses imply that some participants could not be included in the longitudinal analysis. By modelling the remaining missing observations such as deaths explicitly, we were however able to analyse full trajectories for a broad sample of older adults over an extended timeframe, without hidden attrition.

Building on this innovative work, future studies could investigate the time-varying effect of factors associated with healthcare utilization inside specific subgroups. For instance, the occurrence or aggravation of chronic illnesses may explain some of the patterns preceding early deaths and bring valuable insights needed to explore the under-utilization hypothesis. On the intensive utilization side, additional information on the reasons for healthcare consumption would expand the constant high ambulatory care cluster characterization. Indeed, the fact that participants in this trajectory group made repeated emergency visits throughout the follow-up period warrants further in-depth investigation of their attributes and health profiles. Finally, it would be beneficial to compare our results with a similar methodology applied to administrative data or to an international cohort to shed light on the specificities of the longitudinal data analysed in this study.

These results emphasize that a one-size-fits-all approach is not appropriate for delivering care to population segments as diverse as, for instance, individuals living alone and with early onset of frailty (AC2NH cluster), individuals with a moderate functional decline (IHU cluster), and older adults with neither signs of frailty nor functional decline but an intensive healthcare utilization (HAC cluster). Such heterogeneity in healthcare utilization, reflecting a diversity of needs, has to be taken more thoroughly into account when developing targeted and efficient health systems. Utilization behaviours across health services should be important tenets of a comprehensive person-centred care, where they are to complement traditional determinants such as patients’ age or disease status.

## Conclusion

We presented in this paper a novel typology of older adults' healthcare utilization trajectories and described the factors associated with each trajectory group. Identifying distinct healthcare utilization patterns – and the proportion of individuals they represent – can provide an evidence-based and quantitative overview to inform resource planning in the context of a regional health system. An ageing population offers fresh challenges to health services research but current and future studies that address those in a holistic manner – following individuals over a long timespan and encompassing as many care settings as possible – are well-positioned to provide innovative answers.

## Supplementary Information

**Additional file 1.**

## Data Availability

The data and code that support the findings of this study are available from the corresponding author, Leonard Roth, upon reasonable request. Contact: leonard.roth@unisante.ch.
