# Supplementary material for: Identifying common patterns of health services use: a longitudinal study of older Swiss adults' care trajectories
Source: BMC Health Serv Res. 2022 Dec 26;22:1586. doi: 10.1186/s12913-022-08987-z (PMC9793504; doi:10.1186/s12913-022-08987-z)
Supplement: Supplementary file 1 — Additional file 1. [file 12913_2022_8987_MOESM1_ESM.pdf]

Lc65+ study

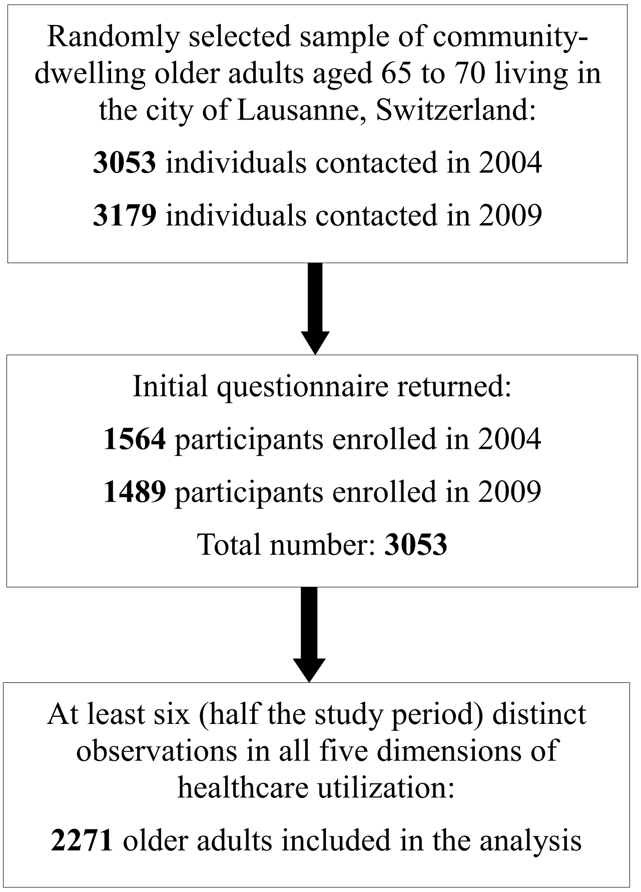

Supplementary Figure 1: Flow diagram of participants included in the analysis.

		Whole sample	Included	Excluded	p-value
**Total N (%)**		3053	2271 (74.4)	782 (25.6)	
**Recruitment wave**	1	51.2%	50.9%	52.3%	0.513
	2	48.8%	49.1%	47.7%	
**Age (years)**	Mean (SD)	67.9 (1.4)	67.9 (1.4)	68.1 (1.4)	0.001
**Sex**	Female	58.7%	60.7%	53.1%	<0.001
	Male	41.3%	39.3%	46.9%	
**Living alone**	No	62.3%	62.8%	60.9%	0.556
	Yes	37.4%	36.9%	38.7%	
	missing	0.3%	0.3%	0.4%	
**Born in Switzerland**	No	29.2%	26.4%	37.6%	<0.001
	Yes	70.6%	73.5%	62.0%	
	missing	0.2%	0.1%	0.4%	
**Difficult financial situation**	No	71.8%	83.0%	39.5%	<0.001
	Yes	11.7%	12.6%	8.8%	
	missing	16.5%	4.4%	51.7%	
**Grip strength**	Normal	72.4%	83.8%	39.3%	<0.001
	Low	10.7%	11.3%	9.0%	
	missing	16.9%	4.9%	51.8%	
**Diagnosed chronic illnesses**	0	12.7%	13.7%	10.1%	0.002
	1	23.7%	23.8%	23.5%	
	2+	63.1%	62.4%	65.3%	
	missing	0.4%	0.2%	1.0%	
**Self-perceived health**	Good	64.8%	70.5%	48.5%	<0.001
	Av	29.2%	25.8%	39.3%	
	Bad	5.6%	3.5%	11.6%	
	missing	0.3%	0.2%	0.6%	
**Difficulties IADLs**	None	87.5%	90.6%	78.3%	<0.001
	Yes without help	6.6%	5.3%	10.4%	
	Yes with help	4.9%	3.3%	9.6%	
	missing	1.0%	0.8%	1.8%	
**Ambulatory care**	none	7.6%	7.4%	8.4%	<0.001
	1 or 2	23.5%	25.4%	18.2%	
	3 to 5	33.1%	33.5%	31.8%	
	6 to 11	21.4%	22.0%	19.4%	
	12+	11.6%	9.9%	16.8%	
	missing	2.8%	1.9%	5.4%	
**Emergency care**	none	72.4%	74.0%	67.5%	<0.001
	once	14.0%	14.6%	12.3%	
	2 or 3	7.2%	6.7%	8.6%	
	4+	1.3%	0.8%	2.7%	
	missing	5.1%	3.8%	9.0%	
**Hospitalisation**	none	79.9%	82.7%	71.7%	<0.001
	once	12.7%	11.8%	15.5%	
	2 or 3	3.5%	2.9%	5.1%	
	4+	0.7%	0.4%	1.5%	
	missing	3.2%	2.2%	6.1%	
**Home care**	no	93.7%	95.6%	88.1%	<0.001
	temporarily	3.0%	2.5%	4.5%	
	regularly	1.8%	1.2%	3.6%	
	missing	1.5%	0.7%	3.8%	

Supplementary Table S1: Comparison of baseline characteristics of included versus excluded individuals (exclusion happened when more than half of the follow-up period was missing). Bivariate relationships are evaluated with chi-squared tests (ANOVA for the age). Ambulatory (emergency) care correspond to the number of annual (emergency) consultations. No individuals were institutionalised at the time of recruitment.

		Increasing healthcare utilization	Late health deterioration	Ambulatory care to nursing home	Early fatal event	High ambulatory care
**Age**	years	**1.23**** (1.01 - 1.49)	**1.09** (0.89 - 1.34)	**2.10***** (1.34 - 3.3)	**1.04** (0.83 - 1.30)	**1.13** (0.84 - 1.53)
**Sex**	male (vs. female)	**1.08** (0.70 - 1.67)	**1.88***** (1.21 - 2.91)	**2.98**** (1.22 - 7.28)	**2.05***** (1.27 - 3.31)	**0.37**** (0.16 - 0.85)
**Living alone**	yes (vs. no)	**1.40** (0.92 - 2.12)	**1.34** (0.86 - 2.10)	**6.29***** (2.48 - 16)	**1.56*** (0.97 - 2.52)	**1.31** (0.70 - 2.48)
**Self-perceived health**	average (vs. good)	**2.15***** (1.37 - 3.36)	**1.24** (0.76 - 2.03)	**1.83** (0.63 - 5.27)	**2.25***** (1.34 - 3.80)	**4.35***** (2.11 - 8.95)
	bad (vs. good)	**2.39*** (0.95 - 5.99)	**1.36** (0.47 - 3.90)	**4.88**** (1.29 - 18.49)	**4.05***** (1.58 - 10.39)	**13.92***** (4.86 - 39.88)
**Grip strength**	low (vs. normal)	**1.5** (0.88 - 2.54)	**1.16** (0.62 - 2.16)	**2.85**** (1.14 - 7.12)	**1.94**** (1.09 - 3.46)	**1.3** (0.62 - 2.76)
**Diagnosed chronic illnesses**	1 (vs. 0)	**1.02** (0.47 - 2.23)	**1.35** (0.65 - 2.80)	NA	**0.66** (0.32 - 1.39)	NA
	2+ (vs. 0)	**1.17** (0.58 - 2.37)	**1.14** (0.57 - 2.26)	NA	**0.63** (0.33 - 1.22)	NA
**Difficulties IADLs**	yes without help (vs. no)	**1.86*** (0.94 - 3.67)	**2.64***** (1.27 - 5.48)	**3.34*** (0.99 - 11.27)	**0.73** (0.24 - 2.19)	**1.47** (0.59 - 3.69)
	yes with help (vs. no)	**2.96**** (1.25 - 6.99)	**3.53**** (1.33 - 9.33)	**25.01***** (8.66 - 72.27)	**3.45***** (1.40 - 8.50)	**1.9** (0.62 - 5.75)

Supplementary Table S2: Results of the multinomial regression model with seven selected explanatory variables (n = 2256). Coefficients are adjusted odds ratios for membership into each cluster compared to the reference one (low healthcare utilization). Confidence intervals in parenthesis are at a 95% level. NAs are present when there is an empty category.
* p < 0.1; ** p < 0.05; *** p < 0.01.

Dissimilarity measure between two sequences

When operating a Sequence Analysis, the discrepancy across all trajectories is summarized into a dissimilarity matrix. In our study, this matrix (dimension 2271*2271) is distributed as follows:

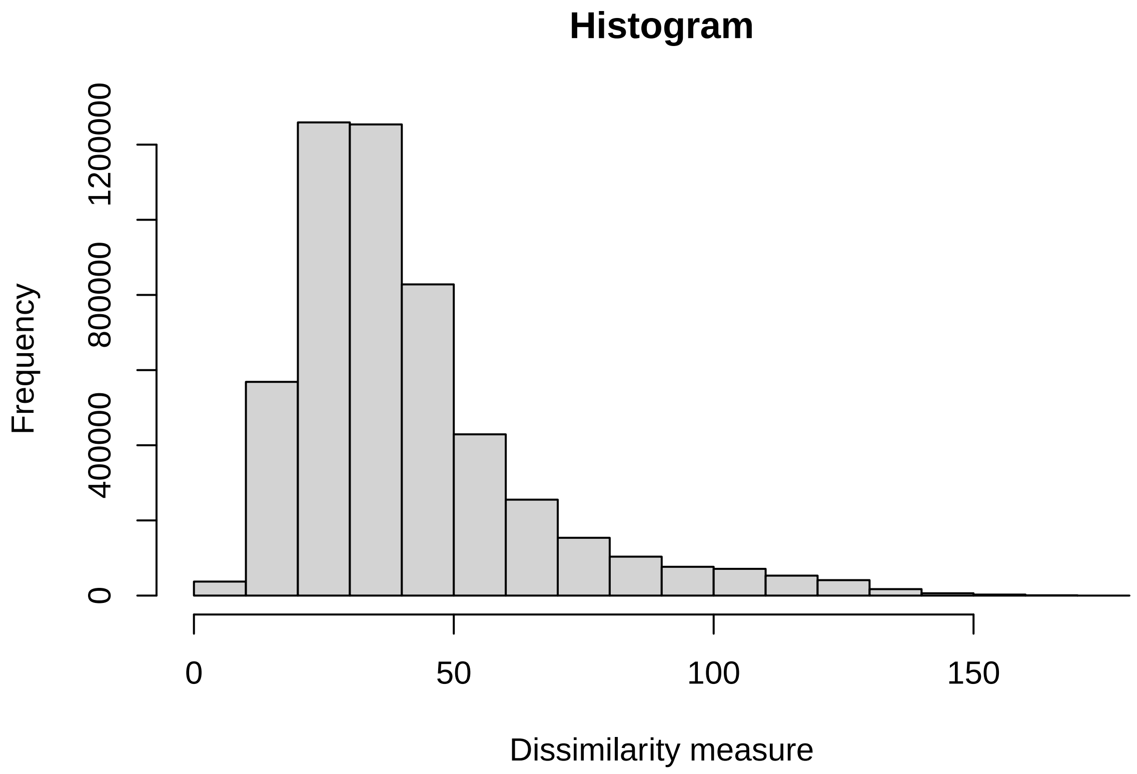

The median value is 35.44. To illustrate how these values are obtained, we will take as example the dissimilarity measure between the two following multidimensional sequences (corresponding to the median distance):

Participant 1

Year	Ambulatory care	Emergency care	Hospitalisation	Home care	Nursing home
1	none	none	none	no	none
2	none	none	none	no	none
3	1 or 2	none	none	no	none
4	1 or 2	none	none	no	none
5	1 or 2	none	none	no	none
6	none	none	none	no	none
7	none	none	none	no	none
8	1 or 2	none	none	no	none
9	none	none	none	no	none
10	6 to 11	none	none	no	none
11	1 or 2	none	none	no	none

Participant 2

Year	Ambulatory care	Emergency care	Hospitalisation	Home care	Nursing home
1	6 to 11	none	none	no	none
2	3 to 5	none	none	no	none
3	3 to 5	none	none	no	none
4	1 or 2	none	none	no	none
5	3 to 5	<NA>	none	no	none
6	3 to 5	once	2 or 3	reg	none
7	6 to 11	none	none	no	none
8	3 to 5	once	once	no	none
9	3 to 5	none	none	temp	none
10	6 to 11	none	once	reg	none
11	1 or 2	none	none	reg	none

Year is year since entry into the study, ambulatory care is number of physician's appointments, emergency care is number of emergency consultations, hospitalisations are overnight stays, home care is professional home care and nursing home is stay of at least one night. 'Temp' stands for temporary and 'reg', for regular.

The first step of a multidimensional Optimal Matching (OM) measure is to set substitution and indel (insertion-deletion) costs. Substitution costs are dimension- or channel-specific and user-defined as follows:

Ambulatory care

	none	1 or 2	3 to 5	6 to 11	12+	incap	dead	*
none	0	1	2	3	4	4	5	1
1 or 2	1	0	1	2	3	3	4	1
3 to 5	2	1	0	1	2	2	3	1
6 to 11	3	2	1	0	1	1	2	1
12+	4	3	2	1	0	1	1	1
incap	4	3	2	1	1	0	1	1
dead	5	4	3	2	1	1	0	1
*	1	1	1	1	1	1	1	0

Emergency care

	none	once	2 or 3	4+	incap	dead	*
none	0	1	2	3	3	4	1
once	1	0	1	2	2	3	1
2 or 3	2	1	0	1	1	2	1
4+	3	2	1	0	1	1	1
incap	3	2	1	1	0	1	1
dead	4	3	2	1	1	0	1
*	1	1	1	1	1	1	0

Hospitalisation

	none	once	2 or 3	4+	incap	dead	*
none	0	1	2	3	3	4	1
once	1	0	1	2	2	3	1
2 or 3	2	1	0	1	1	2	1
4+	3	2	1	0	1	1	1
incap	3	2	1	1	0	1	1
dead	4	3	2	1	1	0	1
*	1	1	1	1	1	1	0

Home care

	no	temp	reg	incap	dead	*
no	0	1	2	2	3	1
temp	1	0	1	1	2	1
reg	2	1	0	1	1	1
incap	2	1	1	0	1	1
dead	3	2	1	1	0	1
*	1	1	1	1	1	0

Nursing home

	none	temp	perm	incap	dead	*
none	0	1	2	2	3	1
temp	1	0	1	1	2	1
perm	2	1	0	1	1	1
incap	2	1	1	0	1	1
dead	3	2	1	1	0	1
*	1	1	1	1	1	0

Channel-specific indel costs are each time half the maximum substitution cost. There are no occurrences of either long common unidimensional or long common multidimensional subsequences in our example so the OM measure is simplified into computing the cost of substituting one sequence by the other in each healthcare utilisation dimension independently. Substitution costs between individual states are thus applied as follows:

Variation between ambulatory care sequences: $3+2+1+0+1+2+3+1+2+0+0=15$.

Variation between emergency care sequences: $1+1+1=3$.

Variation between hospitalisation sequences: $2+1+1=4$.

Variation between home care sequences: $2+1+2+2=7$.

Variation between nursing home sequences: 0.

The first substitution cost (3) corresponds to replacing 'none' by '6 to 11' and applying the ambulatory care substitution cost matrix introduced above. The same principle can be extended to all other computations. The total variation between the two participants is then derived in an additive way, with dimension-specific weights corresponding to the ratio of the maximum substitution costs ($5/5 = 1$ for ambulatory care, $5/4 = 1.25$ for emergency care and hospitalisation, and $5/3 = 1.67$ for home care and nursing home):

Total variation between the trajectories: $1*15+1.25*3+1.25*4+1.67*7+1.67*0=35.44$.
